# The protean manifestations of central nervous system IgG4-related hypertrophic pachymeningitis: a report of two cases

**DOI:** 10.1186/s41016-021-00233-5

**Published:** 2021-02-04

**Authors:** Peter Y. M. Woo, Ben C. F. Ng, June H. M. Wong, Oliver K. S. Ng, Timothy S. K. Chan, Ngai-Fung Kwok, Kwong-Yau Chan

**Affiliations:** 1 Department of Neurosurgery, Kwong Wah Hospital, Hong Kong, Hong Kong; 2 Department of Internal Medicine, Caritas Medical Center, Hong Kong, Hong Kong; 3 Department of Pathology, Kwong Wah Hospital, 25 Waterloo Road, Yaumatei, Hong Kong, Hong Kong; 4 Department of Neurosurgery, Queen Elizabeth Hospital, Hong Kong, Hong Kong

**Keywords:** Hypertrophic pachymeningitis, IgG4-related disease, IgG4-related sclerosing disease, Central nervous system

## Abstract

**Background:**

IgG4-related hypertrophic pachymeningitis is a relative newly recognized and rare manifestation of IgG4-related disease, an immune-mediated fibroinflammatory tumefactive disorder. Fewer than 80 patients have been reported in the literature, and it can mimic common neurosurgical conditions. We describe the clinical presentation of two patients that were initially considered to have a subdural collection, tuberculous meningitis, and a cervical spinal meningioma, but were eventually diagnosed with this disease.

**Case presentation:**

Two ethnic Chinese men, 86 and 62 years old, experienced a 4-week history of headache. Both patients had a history of autoimmune disease, namely glomerulonephritis and Grave’s disease, respectively. Magnetic resonance brain imaging revealed diffuse dural thickening with the latter patient exhibiting homogeneous and intense gadolinium-contrast enhancement. Since the 86-year-old patient also had progressive bilateral visual loss, giant cell arteritis was suspected and a 2-week course of glucocorticoid therapy was prescribed, but his symptoms failed to improve. The 62-year-old patient also had accompanying low-grade fever and was treated empirically as having tuberculous meningitis although there were no confirmatory microbiological findings. This patient further developed right hemiparesis, and additional imaging revealed a C4/5 intradural-extramedullary contrast-enhancing lesion resembling a meningioma causing cord compression. Both patients underwent neurosurgical intervention with the former undergoing a dural biopsy and the latter having the cervical lesion resected. The final diagnosis was IgG4-related hypertrophic pachymeningitis with the hallmark histological features of lymphoplasmacytic infiltration of IgG4+ plasma cells, storiform fibrosis, and obliterative phlebitis. In addition, their serum IgG4 levels were elevated (i.e., > 135 mg/dL). Both patients received at least 6 months of glucocorticoid therapy while the latter also had azathioprine. Their symptoms improved significantly and recurrent lesions were not detected on follow-up imaging.

**Conclusions:**

A high index of suspicion for this condition is suggested when a male patient with a history of autoimmune disease and compatible radiological findings, experiences subacute headache that is disproportionate to the degree of dural involvement. Neurosurgeons should consider early meningeal biopsy to establish a definitive histological diagnosis in order for early effective immunosuppressive treatment to be initiated and to avoid unnecessary morbidity.

## Background

IgG4-related disease (IgG4-RD), also known as IgG4-related sclerosing disease, is a rare immune-mediated fibroinflammatory condition with unique clinical, serological, and pathological features. It can involve any organ system and has the tendency to form multiple tumefactive lesions. IgG4-RD was initially identified in the pancreas (autoimmune pancreatitis), but has gradually been diagnosed at other sites such as the bile ducts (sclerosing cholangitis), the salivary glands (sclerosing sialadenitis), breast, kidneys, thyroid, and prostate glands [1, 2]. The most common central nervous system (CNS) manifestation of IgG4-RD is hypophysitis followed by hypertrophic pachymeningitis (HPM) [3]. From a Japanese national survey, the crude prevalence of HPM was 0.95 cases per 100,000 with only 8.8% due to IgG4-RD [4]. In the last decade, fewer than 80 patients have been described in the literature since our first reported case [5]. The presentation of IgG4-related HPM can be protean and could mimic neoplastic, chronic inflammatory, infectious, or hemorrhagic conditions [3]. We present our experience in managing two patients with IgG4-related HPM and emphasize the importance of confirming a histological diagnosis to avoid unnecessary empirical treatment and allow for timely initiation of immunosuppressive therapy.

## Case presentation

### Patient 1

An 86-year-old man presented with spontaneous progressive visual loss and diffuse headache for 4 weeks. He had a history of asthma and perinuclear anti-neutrophil cytoplasmic antibodies-associated glomerulonephritis. Clinical examination revealed significantly impaired bilateral visual acuity (right eye could not perceive light; left eye was 0.016). There was also a right relative afferent pupillary defect and fundoscopy revealed bilateral optic atrophy. There was no focal scalp tenderness.

Serum laboratory tests revealed mild neutrophilic leukocytosis of 11.4 × 10^9^/L with an elevated erythrocyte sedimentation rate (ESR) of 92 mm/L and c-reactive protein (CRP) level of 25 mg/L. Lumbar cerebrospinal fluid (CSF) sampling showed lymphocytic pleocytosis with a white cell count (WCC) of 32/mm^3^ (84% lymphocytes) with a slightly elevated protein level (1.16 g/L) and normal glucose levels (4.1 mmol/L).

The patient was initially empirically managed as having giant cell arteritis and pulse prednisolone therapy (50 mg daily) was prescribed. A superficial temporal artery biopsy performed the next day showed no evidence of inflammation.

**Fig. 1:**
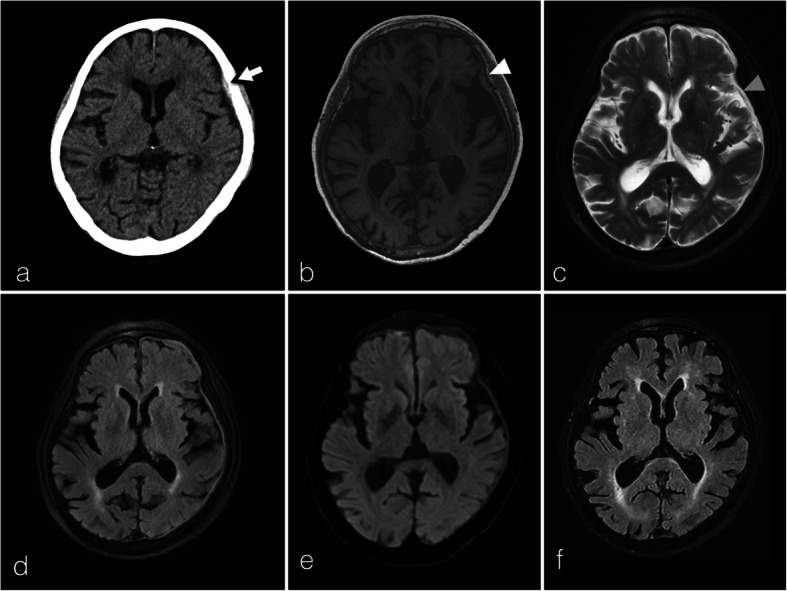
Patient 1. Axial non-contrast-enhanced CT brain scan revealing a left frontal convexity region hyperdense extra-axial lesion resembling an acute subdural hematoma (**a**, white arrow). A non-contrast-enhanced MRI scan exhibiting a T1-weighted isointense (**b**, white arrowhead) and T2/FLAIR-weighted hypointense left hemispheric pachymeningeal lesion with areas of hyperintense signal changes representing inflammatory foci (**c**, T2W, grey arrowhead; **d**, FLAIR sequence). The lesion did not show evidence of restricted diffusion (**e**). Three months after initiating prednisolone, there was complete resolution of the pachymeningeal lesion (**f**, FLAIR sequence)

A non-contrast-enhanced computed tomography (CT) brain scan revealed a left frontal thin 3 mm hyperdense extra-axial lesion that resembled an acute subdural hematoma (Fig. 1a). Due to the patient’s chronic renal impairment, only a non-contrast-enhanced magnetic resonance imaging (MRI) scan could be performed. There was a diffuse left hemispheric T1-weighted sequence isointense and T2/FLAIR-weighted sequence hypointense pachymeningeal lesion with areas of hyperintense signal changes (Fig. 1b–d). There was no evidence of restricted diffusion on diffusion-weighted imaging (Fig. 1e). The initial radiological impression was a subdural hematoma although the patient had no history of head trauma, and there was no evidence to suggest he had a bleeding tendency.

**Fig. 2:**
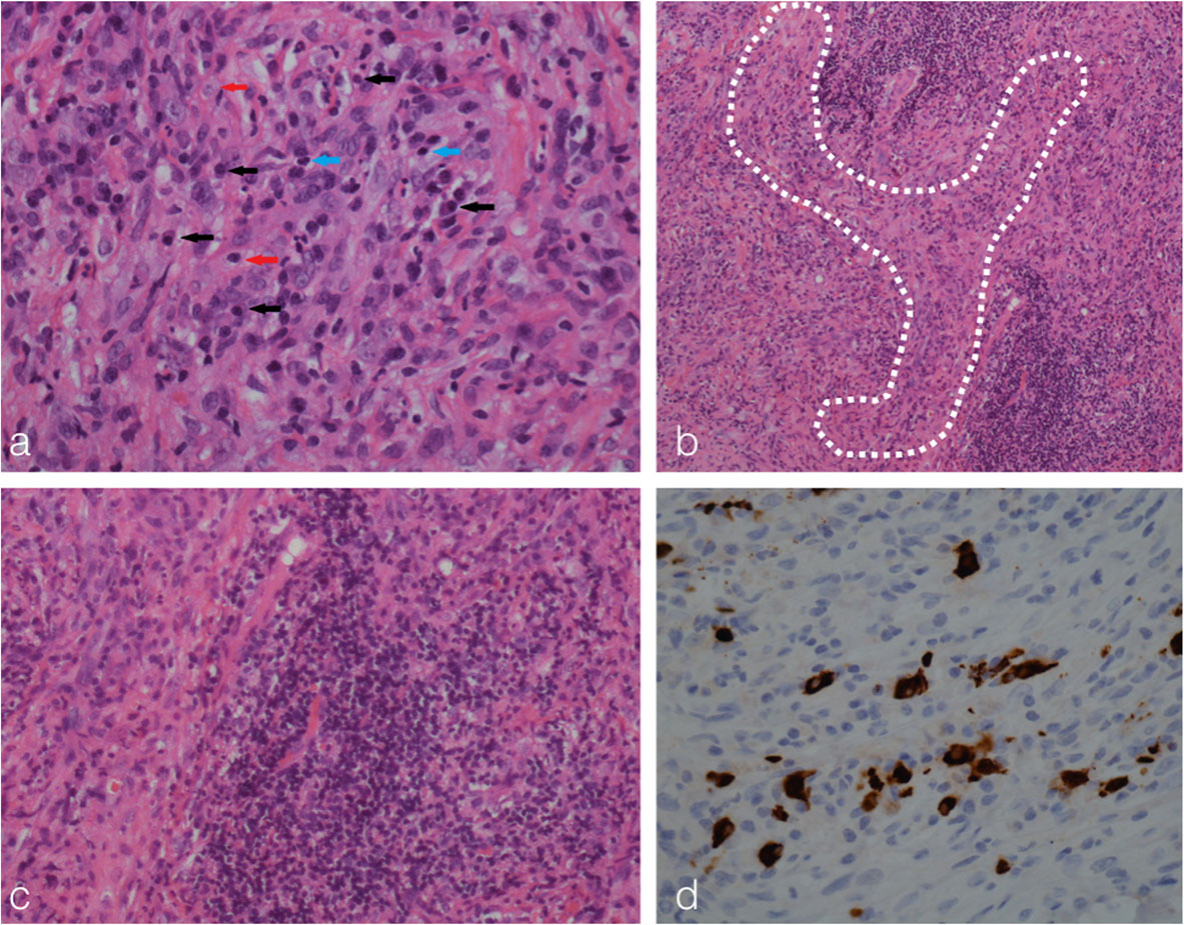
Patient 1. Histopathological microphotographs of the dural lesion showing classical features of IgG4-RD. Diffuse lymphoplasmacytic infiltrates comprising of plasma cells (black arrow), lymphocytes (blue arrow), and histiocytes (red arrow) (**a**, hematoxylin and eosin stain, HPF). Storiform “cartwheel” fibrosis was observed (**b**, white-dotted area, hematoxylin and eosin stain, LPF). Obliterative phlebitis was also detected (**c**, Hematoxylin and Eosin stain). Additional IHC showed > 10 IgG4+ plasma cells per HPF (**d**)

A burr hole procedure was performed 1 week after starting pulse steroids and white fibrotic thickened dura was encountered without the presence of subdural hemorrhage. Histological examination of the dural biopsy revealed chronic lymphoplasmacytic infiltrates, storiform fibrosis, and obliterative phlebitis (Fig. 2a–c). Immunohistochemical (IHC) staining showed 20 IgG4+ (immunoglobulin-G4) plasma cells per high power field (HPF) with an IgG4/IgG ratio of 30% (Fig. 2d). The patient’s serum IgG4 level was subsequently noted to be elevated at 202 mg/dL (normal range 8–140 mg/dL).

A diagnosis of IgG4-related HPM was established and a 2-week course of oral prednisolone (50 mg daily) was started with subsequent tapering to 2.5 mg daily over 6 months. One month after starting glucocorticoid therapy, the patient’s visual acuity improved (right 0.016, left 0.1). A 3-month MRI scan showed resolution of the dural thickening (Fig. 1f) and serum IgG4 levels were normalized.

### Patient 2

**Fig. 3:**
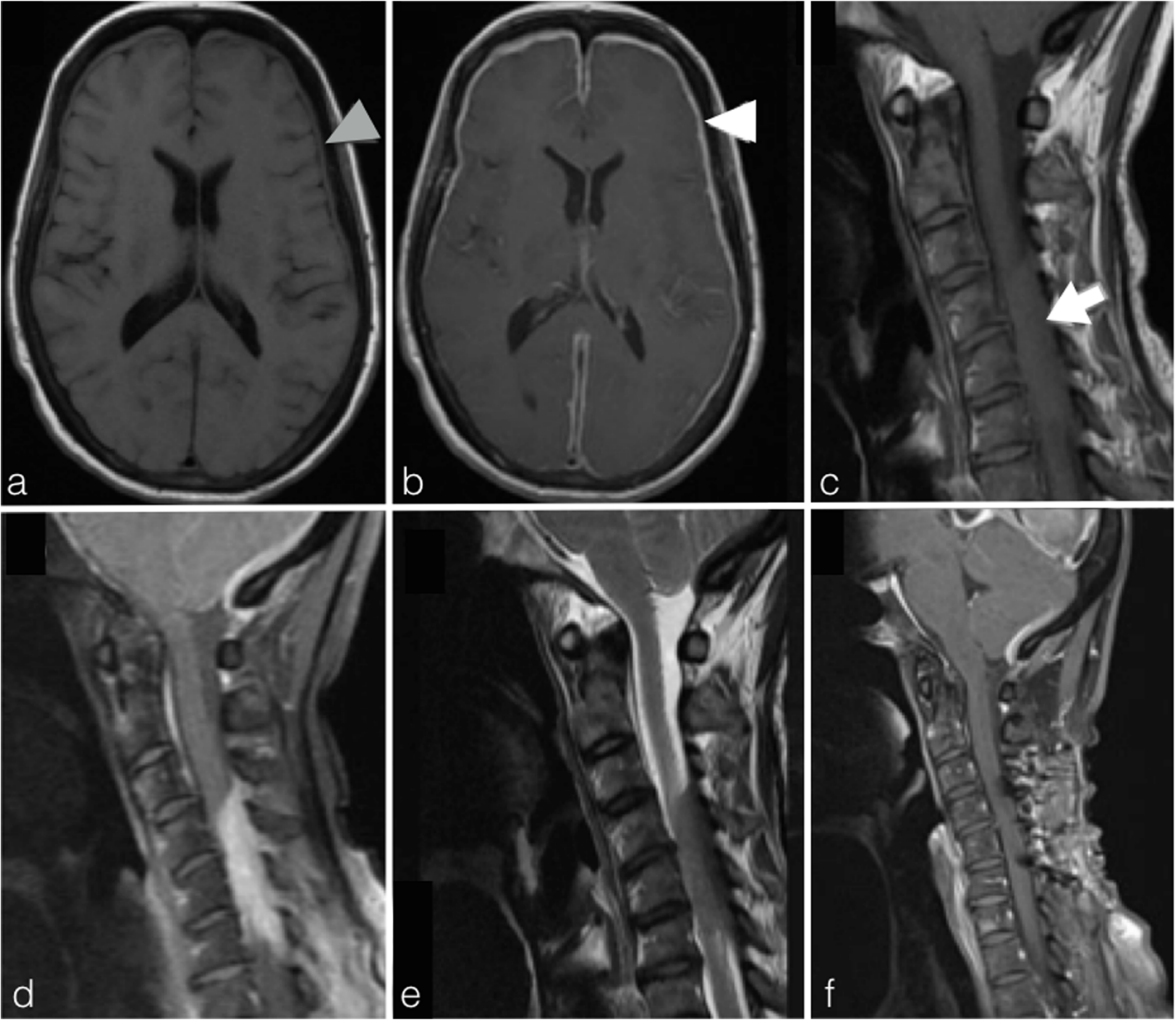
Patient 2. MRI brain scans revealed diffuse bilateral pachymeningeal thickening that was T1-weighted isointense (**a**, axial, grey arrowhead) and demonstrated intense contrast-enhancement (**b**, white arrowhead). Cervical spinal imaging also revealed an intradural-extramedullary lesion at the C4/5 level posterior thecal sac that was also T1-weight isointense (**c**, sagittal, white arrow) and exhibited homogeneous contrast enhancement with a dural-tail sign (**d**). The spinal lesion caused significant cord compression (T2W, sagittal). After 2 months of prednisolone and azathioprine, a repeat MRI scan showed no recurrent lesion (**f**, contrast-enhanced T1W)

A 62-year-old man, with a history of Grave’s disease, presented with gradual onset headache and low-grade fever for 4 weeks. Clinical examination showed no nuchal rigidity and there was no neurological deficit. A gadolinium contrast-enhanced MRI brain scan showed diffuse bilateral leptomeningeal thickening with contrast enhancement (Fig. 3a, b). Lumbar puncture-derived CSF samples showed a raised WCC (40/mm^3^) and protein levels (1.8 g/L) whereas the glucose level was normal (3.0 mmol/L). The patient was empirically medically treated as having mycobacterium tuberculosis meningitis (TBM), which is endemic in South East Asia, with rifampicin, pyrazinamide, isoniazid, levofloxacin, and prednisolone although no microbiological investigations, including CSF specimens, could confirm this diagnosis. After initiating treatment, her headache improved slightly and she became afebrile.

Before follow-up imaging could be performed, the patient developed a 2-week history of right-sided weakness with neck pain 3 months after starting empirical anti-TB treatment. Physical examination showed right hemiparesis (Medical Research Council grade 4/5) with signs of cervical myelopathy. A MRI cervical spine showed a C4-5 level homogeneous contrast-enhancing intradural-extramedullary nodular mass exhibiting a dural tail sign causing spinal cord compression (Fig. 3c–e). The presumptive diagnosis was a cervical meningioma and a C4/5 laminoplasty with excision of the lesion was performed. Intraoperatively, a grey-white fibrous avascular dural mass was encountered and gross total resection was performed. Postoperatively, the patient experienced significant recovery of her hemiparesis. Histological examination of the spinal lesion demonstrated a fibrous tissue mass with lymphoplasmacytic infiltrates and storiform fibrosis. IHC staining showed an elevated IgG4 + plasma cell count of 32/HPF and the IgG4/IgG ratio was 80%.

A tapering course of oral prednisolone starting from 50 mg to 10 mg was prescribed over a course of 8 months. Azathioprine 75 mg was subsequently started as a steroid-sparing agent. There was a 37% reduction of the patient’s serum IgG4 level from 270 mg/dL to 171 mg/dL during this period. A MRI scans of both the brain and cervical spine performed 2 months after glucocorticoid and azathioprine treatment showed no significant residual lesion (Fig. 3f).

## Discussion

IgG4-RD is an immune-mediated fibroinflammatory condition that can affect multiply organ systems with an estimated annual incidence of 0.28–1.08 per 100,000 [2]. HPM is a rare subgroup of this disorder with only 2.4% of patients with CNS IgG4-RD presenting with this condition [6]. In contrast to other autoimmune conditions, IgG4-related HPM generally occurs in men (female: male ratio of 1:5.9) in their fifth to sixth decade [2, 4]. The clinical presentation is protean ranging from diffuse symptoms such as chronic headaches, neck stiffness, cognitive decline, and seizures to focal mechanical symptoms due to nerve compression including optic neuropathy, cranial nerve palsies, and cortico-spinal sensorimotor deficits [3, 7]. As in our second case, spinal HPM occasionally requires neurosurgical decompression due to their diffuse extent of disease within the confined space of the spinal canal [8, 9]. A retrospective review of 33 IgG4-related HPM cases observed that two-thirds of patients experienced headache, a third had cranial nerve palsies, and a fifth had visual disturbances [7]. Systemic involvement was noted in 48% of patients mostly involving the bone (12%) followed by the salivary glands (9%) and lungs (9%) [7].

There are four subclasses of human IgG, the most common type of antibody, and IgG4 is the least abundant subclass constituting < 5% of IgG in healthy individuals [10]. IgG4 is recognized as an anti-inflammatory antibody that is believed to attenuate allergic responses by inhibiting IgE-mediated hypersensitivity reactions [10]. The pathogenesis of IgG4-RD remains to be elucidated, but it is currently understood to be an antigen-driven disease involving IgG4+ B cells and CD4+ cytotoxic T cells [2, 6]. Why B cells are clonally expanded and differentiated to IgG4-producing plasma cells remains unknown, but the prevailing hypothesis seems to involve autoimmunity to an unidentified antigen [2, 3]. This results in the activation of CD4+ cytotoxic T cells and the subsequent induction of inflammation and fibroblast activation [1]. It has also been postulated that since IgG4 is predominantly anti-inflammatory in nature and pivotal in immune tolerance, its elevated expression may not be the primary driver for this disease, but a secondary responding event to attenuate CD4+ T cell activity [1, 7].

Although HPM is rare, our experience indicates that it can mimic conditions commonly encountered in daily neurosurgical practice due to its tumefactive nature [11]. Clinical suspicion should be raised when a patient with a strong history of autoimmune disease, as observed in the present two cases, experience a disproportionately severe subacute headache relative to the minimal mass effect elicited by the dural lesion reflecting the inflammatory nature of the disease. The differential diagnoses include intracranial hypotension, leptomeningeal carcinomatosis, subdural empyema, or other causes of pachymeningitis such as TBM. In most circumstances, with the exception of IgG4-RD, these conditions can be readily excluded clinically. Our second patient was first suspected to have a cervical meningioma due to its suggestive radiological features. But only 15% of spinal meningiomas arise in this region with most based at the anterior thecal sac [12]. Alternatively, a diagnosis of tuberculous granuloma was considered given the patient’s previous history of suspected TBM. However, a review of his initial MRI brain scan showed no evidence of associated characteristic features such as basal cisternal involvement or intraparenchymal granulomas. In IgG4-related HPM, lesions are typically avidly contrast-enhancing and on T2-weighted MRI sequences may exhibit hypointense dural thickening with intralesional hyperintense foci representing areas of inflammation which are more evident on fluid-attenuated inversion recovery (FLAIR) sequences [13].

**Fig. 4:**
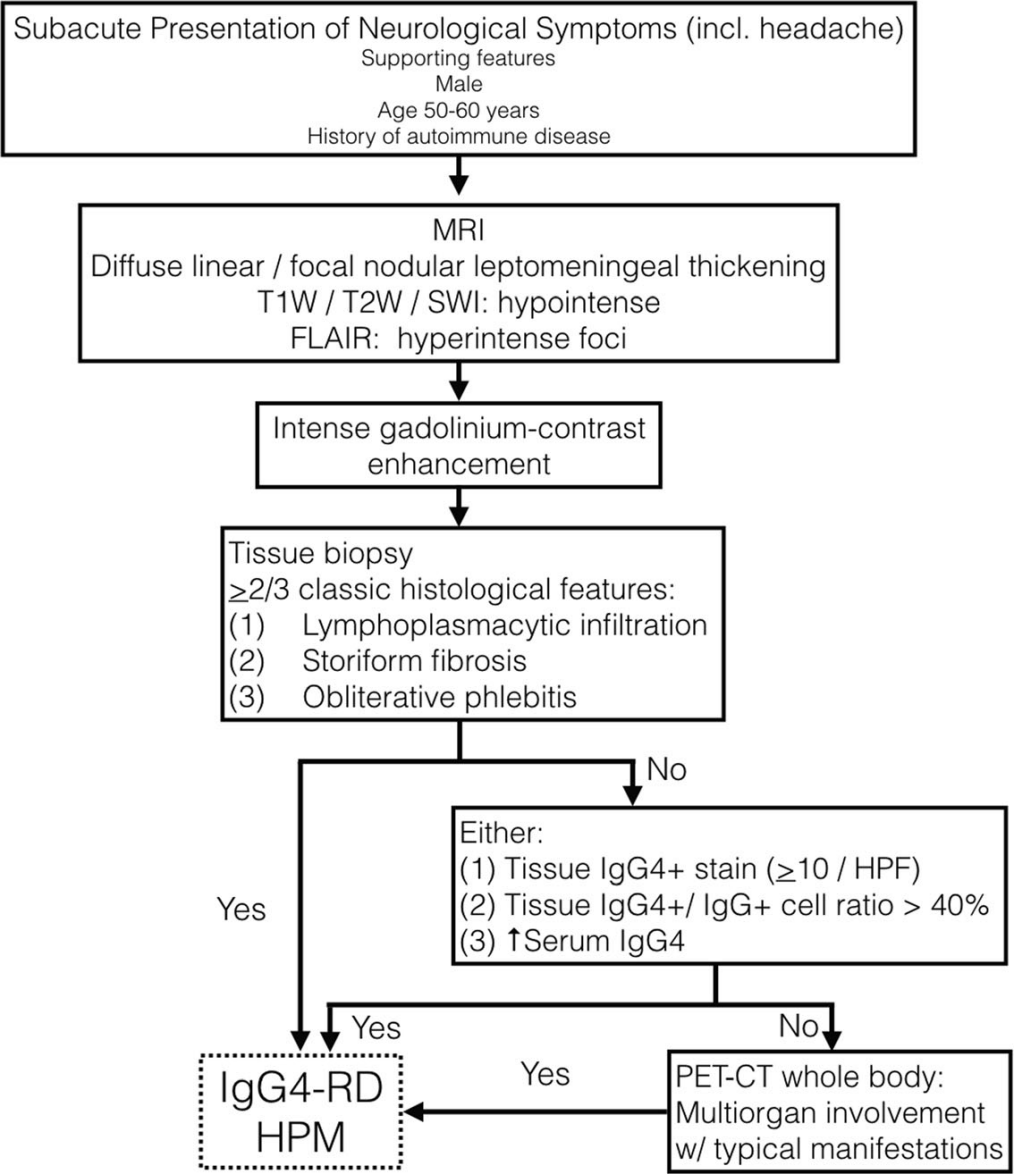
Proposed flowchart delineating the diagnostic process for confirming IgG4-RD HPM in suspected patients. N.B. SWI, susceptibility-weighted imaging; FLAIR, fluid-attenuated inversion recovery

A definitive diagnosis of IgG4-RD requires the fulfillment of clinical and pathological criteria proposed by an international, multidisciplinary consortium in 2011 [14]. The latter requires the presence of two of three classic morphological histologic features, namely dense lymphoplasmacytic infiltrates, storiform fibrosis, and obliterative phlebitis in conjunction with a positive IgG4 stain [14]. The cutoff IgG4+ plasma cell count required for HPM is 10 per HPF and a IgG4+/IgG+ cell ratio of > 40% [14, 15]. When only one histologic feature is identified, supportive evidence of an elevated serum IgG4 level (> 135 mg/dl), which is present in 70–90% of patients with active disease, and demonstration of multi-organ involvement, by either radiological or pathological examination, will be required [7, 14]. We therefore propose a diagnostic algorithm for clinicians to adopt in confirming IgG4-RD HPM in suspected patients (Fig. 4).

There is no consensus on the treatment of IgG4-related HPM, but initial therapy generally involves a 2- to 4-week course of prednisolone (0.6 mg/kg/day) followed by a 3- to 6-month tapering to a daily maintenance dose of 2.5–5.0 mg for up to 3 years [3, 7]. According to a systematic review of 1 220 IgG4-RD patients, 97% responded to glucocorticoid monotherapy [2]. Therapeutic efficacy with steroid-sparing immunosuppressive agents has also been observed, especially during disease relapse [2]. Agents such as azathioprine, mycophenolate mofetil, and methotrexate have been utilized with treatment response rates of 72–100% [2, 3, 7]. Rituximab, an anti-CD20+ B cell monoclonal antibody, has been discovered to be a promising treatment resulting in rapid clinical, radiological, and serological responses [16]. However, due to its high molecular weight < 1% of systemic rituximab crosses the blood-brain barrier. To address this, Della-Torre et al. reported the successful treatment of IgG4-related HPM by administrating the agent intrathecally with minimal adverse effects [17].

## Conclusions

Neurosurgeons should be cognizant of the clinical presentation of IgG4-related HPM as they often present with symptoms that can mimic subdural collections, TBM, or meningiomas. A high-index of suspicion is warranted when male patients with a history of autoimmune disease complain of subacute headache disproportionate to the extent of dural involvement. Although elevated serum IgG4 levels may be supportive, one should consider early meningeal biopsy in order to establish a definitive histologic diagnosis, avoid unnecessary empirical treatment, and to allow for the administration of immunosuppressive therapy.

## Data Availability

Data sharing not applicable to this article as no datasets were generated or analyzed during the current study.

## Abbreviations

CNS: Central nervous system
CRP: C-reactive protein
CSF: Cerebrospinal fluid
CT: Computed tomography
ESR: Erythrocyte sedimentation rate
HPF: High power field
HPM: Hypertrophic pachymeningitis
IHC: Immunohistochemical
IgG: Immunoglobulin-G4
IgG4-RD: IgG4-related disease
MRI: Magnetic resonance imaging
TBM: Tuberculous meningitis
WCC: White cell count
